# Volumetric evaluation of effects of platelet-rich fibrin and concentrated growth factor on early bone healing after endodontic microsurgery: a randomized controlled trial

**DOI:** 10.1186/s12903-023-03530-w

**Published:** 2023-10-29

**Authors:** Jae-Seek You, Gyeo-Woon Jung, Ji-Su Oh, Seong-Yong Moon, Won-Pyo Lee, Hyoung-Hoon Jo

**Affiliations:** 1 Department of Oral and Maxillofacial Surgery, School of Dentistry, Chosun University, Gwangju, Korea; 2 Department of Periodontology, School of Dentistry, Chosun University, Gwangju, Korea; 3 Department of Conservative Dentistry, School of Dentistry, Chosun University, 309 Phimun-daero, Dong-gu, Gwangju, 61452 Korea

**Keywords:** Endodontic microsurgery, Bone defect, Cone-beam computed tomography, Platelet-rich fibrin, Concentrated growth factor

## Abstract

**Background:**

This randomized controlled clinical trial compared the effects of platelet-rich fibrin (PRF) and concentrated growth factor (CGF) on early bone healing after endodontic microsurgery.

**Methods:**

Eighteen patients with an isolated periapical lesion < 10 mm in the maxillary anterior region were randomly assigned to three groups: control, PRF, or CGF. Endodontic microsurgery was performed and PRF or CGF membranes were placed over the bone defects in the experimental groups. The volume of the bone defect at postoperative one week, three months, and six months was evaluated using cone-beam computed tomography and Mimics software. The results were statistically analyzed using the Kruskal–Wallis test and post-hoc Mann–Whitney U test with Bonferroni correction.

**Results:**

At the three-month follow-up, the PRF and CGF groups showed significantly greater bone healing compared with the control group (p > 0.05). However, no significant difference was observed between the PRF and CGF groups. At the six-month follow-up, no significant differences were observed between the groups.

**Conclusions:**

These results suggested that PRF and CGF promote early bone healing after endodontic microsurgery.

## Background

Substances that promote bone formation, including transforming growth factor, platelet-derived growth factor, and bone morphogenetic protein, which are abundant in the blood, are widely used in dentistry to repair bone defects [1]. With the advancement in techniques for preparing blood concentrates, substances including platelets, growth factors, and complex fibrin matrices of various concentrations have been developed [2]. Platelet-derived materials are produced by centrifugation of blood, which is an effective and easy way to obtain growth factors.

Platelet-rich plasma (PRP) is a first-generation platelet-derived material with wound-healing properties [3]. However, a double centrifugation process is required for preparing PRP, and the addition of heterothrombin and anticoagulants may cause immune and infectious reactions [4]. Therefore, currently, PRP is rarely used.

Platelet-rich fibrin (PRF) reported by Chouckroun et al. [5] is a second-generation platelet-derived material that contains more growth factors than PRP. Unlike PRP, PRF is produced by a single centrifugation of autologous blood without the addition of anticoagulants or thrombin and three layers are obtained: RBC layer, Fibrin clot layer (PRF), and serum layer (PPP) [5]. PRF obtained through the gradual coagulation process has a three-dimensional structure with higher strength than natural fibrin coagulation. It has a highly elastic matrix structure that allows the continuous release of various growth factors for a longer time [6]. Fibrin acts as a biological adhesive that allows the stabilization of the initial platelet cluster during coagulation [7].

Concentrated growth factor (CGF) was first prepared by Sacco in 2006 [8]. Similar to PRF, CGF can be obtained without the addition of biochemical agents, which prevents immune responses, toxicity, and cross-contamination. The centrifuge to obtain CGF (Medifuge, Silfradent Srl, Italy) is specially designed such that the centrifugation rate varies with time [8]. The centrifugation process obtains four layers: RBC layer, GF and stem cell layer (CGF), Buffy coat layer, and serum layer (PPP), and produces a dense fibrin matrix with abundant growth factors compared to PRF [9, 10]. Increased cohesion by fibrinogen, factor XIII, and thrombin protects fibrin against plasmin degradation, increasing its tensile strength and stability [11]. CGF has structural properties similar to those of PRF, but it has a more complex three-dimensional fiber structure and contains more growth factors [10, 12].

Platelet-derived materials, including PRF and CGF, contain growth factors such as fibroblast growth factor, vascular endothelial growth factor, insulin-like growth factor, transforming growth factor β, and epidermal growth factor. PRF and CGF are platelet concentrates collected from a single fibrin membrane and contain blood components beneficial for immunity and healing. These substances release growth factors and cytokines that stimulate bone and soft-tissue healing. In addition, the fibrin matrix is responsible for immune regulation and angiogenesis [10, 13]. PRF and CGF are used either alone or in combination to promote soft- and hard-tissue regeneration in dentoalveolar and maxillofacial surgeries [7].

Although favorable outcomes are usually achieved with root canal treatment, symptoms may persist or recur in approximately 10–15% of cases [14]. Cases with unresolved apical periodontitis after nonsurgical endodontic therapy or retreatment are indicated for endodontic microsurgery [15]. The success rate of surgical endodontics has increased with the introduction of microscopes, ultrasonic instruments, micro-instruments, and the development of bioceramic filling materials [16]. Recently, interest in the use of platelet-derived materials to promote bone-defect healing in endodontic microsurgery is increasing [11]. However, few clinical studies have used PRF or CGF alone without bone grafts in surgical endodontics and investigated its efficacy.

This study aimed to evaluate the effects of platelet concentrates on bone regeneration by applying them to bone defects following endodontic microsurgery. The study investigated 2 hypotheses. The first hypothesis suggested that the PRF and CGF promote early healing of bone defects after endodontic microsurgery. The second hypothesis suggested that there is no difference between PRF and CGF in promoting early healing of bone defects after endodontic microsurgery.

## Methods

This study was approved by the Institutional Review Board (IRB) of the Chosun University Dental Hospital (CUDHIRB 1902 010 R01), and the study was registered on cris.nih.go.kr. (registration number: KCT0008664).

### Patient selection

This randomized controlled trial was conducted on patients over 16 years old who visited the Chosun University Dental Hospital from October 2019 to September 2021 for treatment of a periapical lesion. Patients with an isolated periapical lesion in the maxillary anterior region and a lesion size of less than 10 mm (mesiodistal diameter) were included. Patients with systemic diseases, such as diabetes and osteoporosis that could have affected bone metabolism and healing were excluded. Teeth with immature root apex, unrestorable crowns, grade 3 mobility, or fractured roots were also excluded. Informed consent was acquired from all participants after carefully explaining the possible risks and benefits.

The participants were randomly divided into either the control or two experimental groups (PRF or CGF). Randomization was performed using opaque envelopes containing concealed assignment codes with a 1:1:1 randomization allocation ratio. One of the research associates picked up an envelope before surgery and the patients were assigned into each group.

**Fig. 1:**
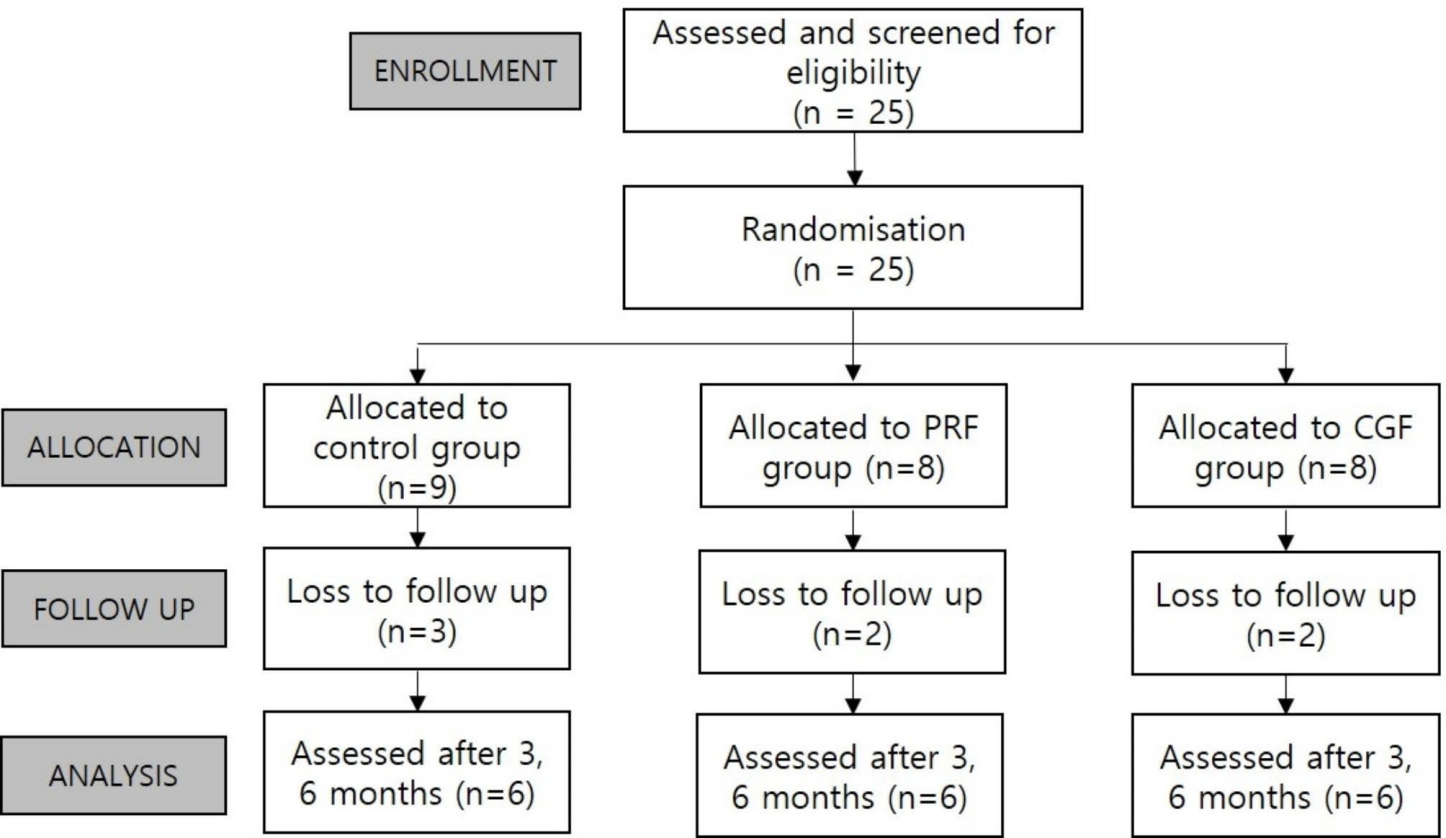
Flowchart of participants through each stage of the trial

### PRF and CGF preparation

**Fig. 2:**
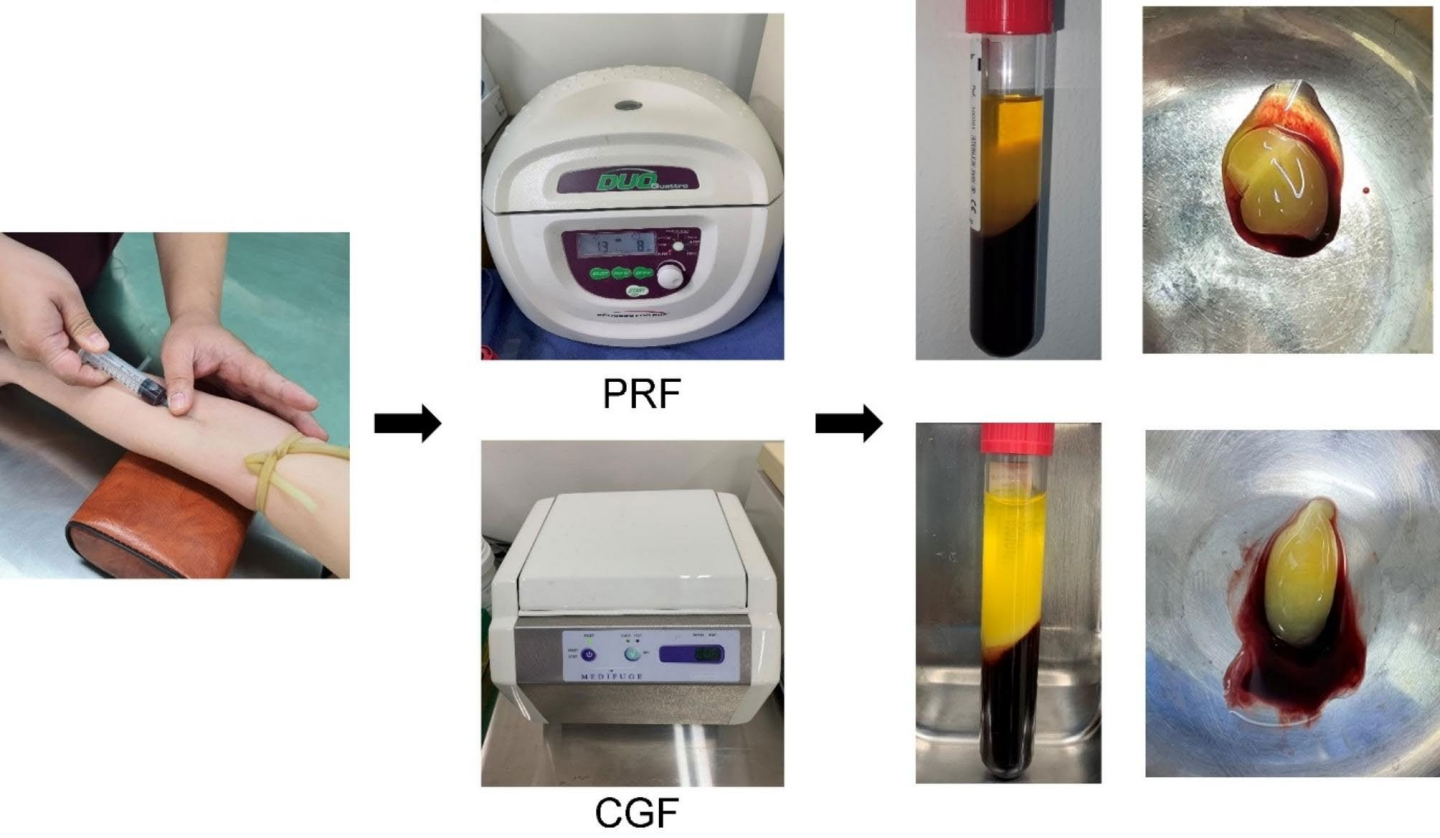
Preparation of platelet-rich fibrin and concentrated growth factor

In the experimental groups, PRF or CGF was prepared preoperatively from 10 mL of venous blood collected from the patient’s forearm, according to the manufacturer’s protocol (Fig. 2). The PRF was prepared following the protocol described by manufacturer instructions at 1,300 rpm for 8 min (DUO Quattro; A-PRF Process, Nice, France). And the CGF was prepared following the protocol described by manufacturer instructions (Medifuge; Silfradent, S. Sofia, Italy): acceleration for 30 s, 2700 rpm for 2 min, 2400 rpm for 4 min, 2700 rpm for 4 min, 3000 rpm for 3 min, deceleration for 36 s and stopped.

### Surgical procedure

**Fig. 3:**
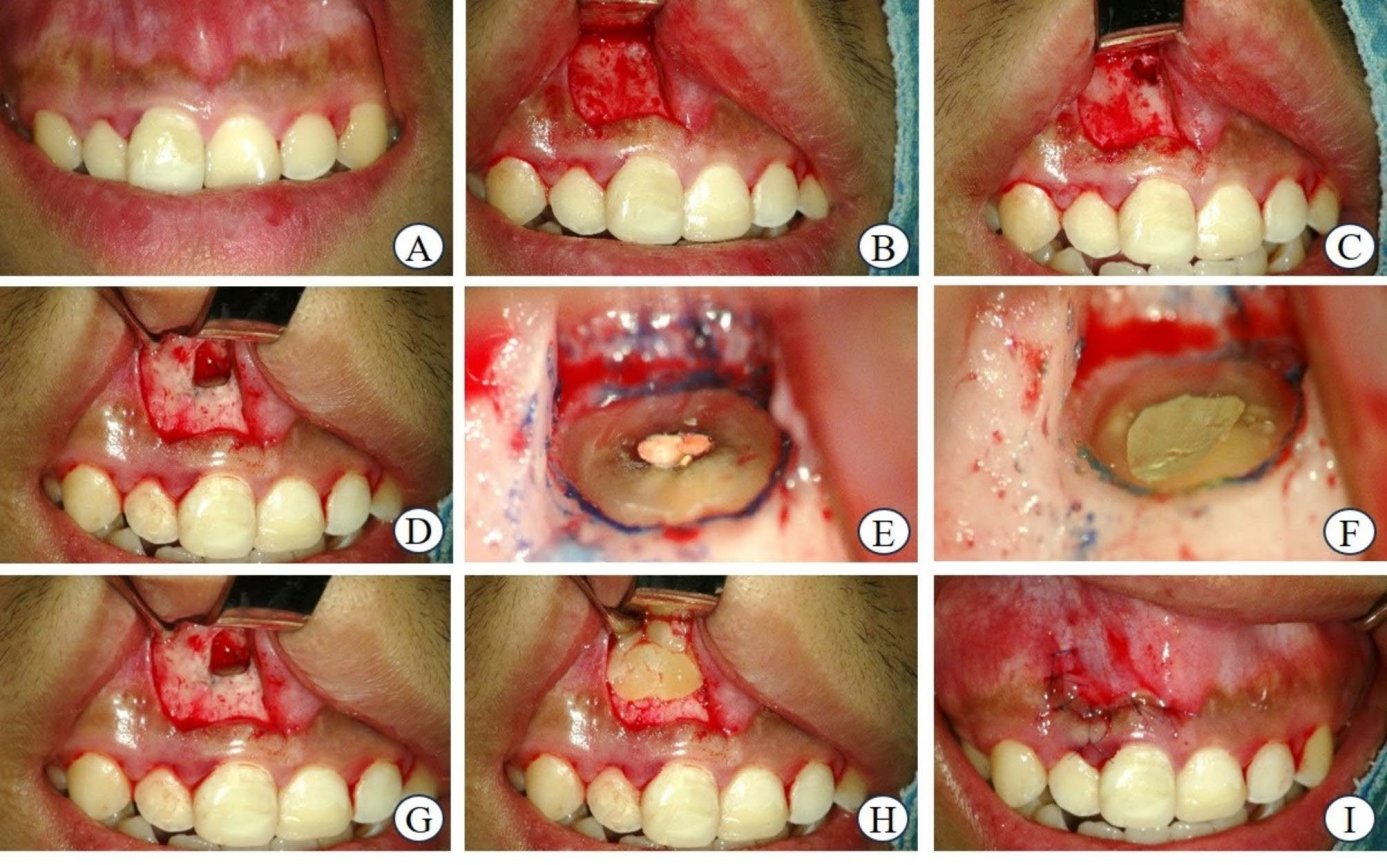
Surgical procedure **A** Preoperative photograph **B** Submarginal incision and full thickness periosteal flap **C** Labial bone osteotomy **D** Curettage and root-end resection **E** Resected surface with methylene blue stain (X20) **F** Retrograde filling (X20) **G** without membrane (control group) **H** platelet-rich fibrin or concentrated growth factor membrane placed over bone defect. **I** Postoperative photograph

All surgeries were performed under local anesthesia (2% lidocaine hydrochloride with 1:100,000 epinephrine) by a single operator using modern microsurgical techniques under a dental microscope (OPMI pico; Carl Zeiss, Gottingen, Germany). After flap elevation, an osteotomy was performed using rotary instruments to expose the root apex. The inflamed tissue was debrided using hand instruments. The apical 3 mm of the root end was resected using a high-speed bur and a 3-mm root-end cavity was prepared using an ultrasonic tip (JETip; B&L Biotech, Ansan, Korea). The retrograde filling was performed using a calcium silicate-based cement (Endocem; Maruchi, Wonju, Korea). In the experimental groups, the apical bone defects were covered with the respective materials in the form of membranes (PRF or CGF). The same surgical procedure was performed in the control group, except that no additional membrane was used. Finally, the flap was replaced and sutured using 6 − 0 nylon (Fig. 3). The sutures were removed one week after surgery.

### Radiographic evaluation

To compare the size of the bone defects, cone-beam computed tomography (CBCT) was performed at 1 week, 3 months, and 6 months postoperatively using a CS9300 3D CBCT unit (Carestream Health Inc., Rochester, NY, USA) with a set value of 4–10 mA and 85–120 kV, and a voxel size of 0.2–0.3 mm.

**Fig. 4:**
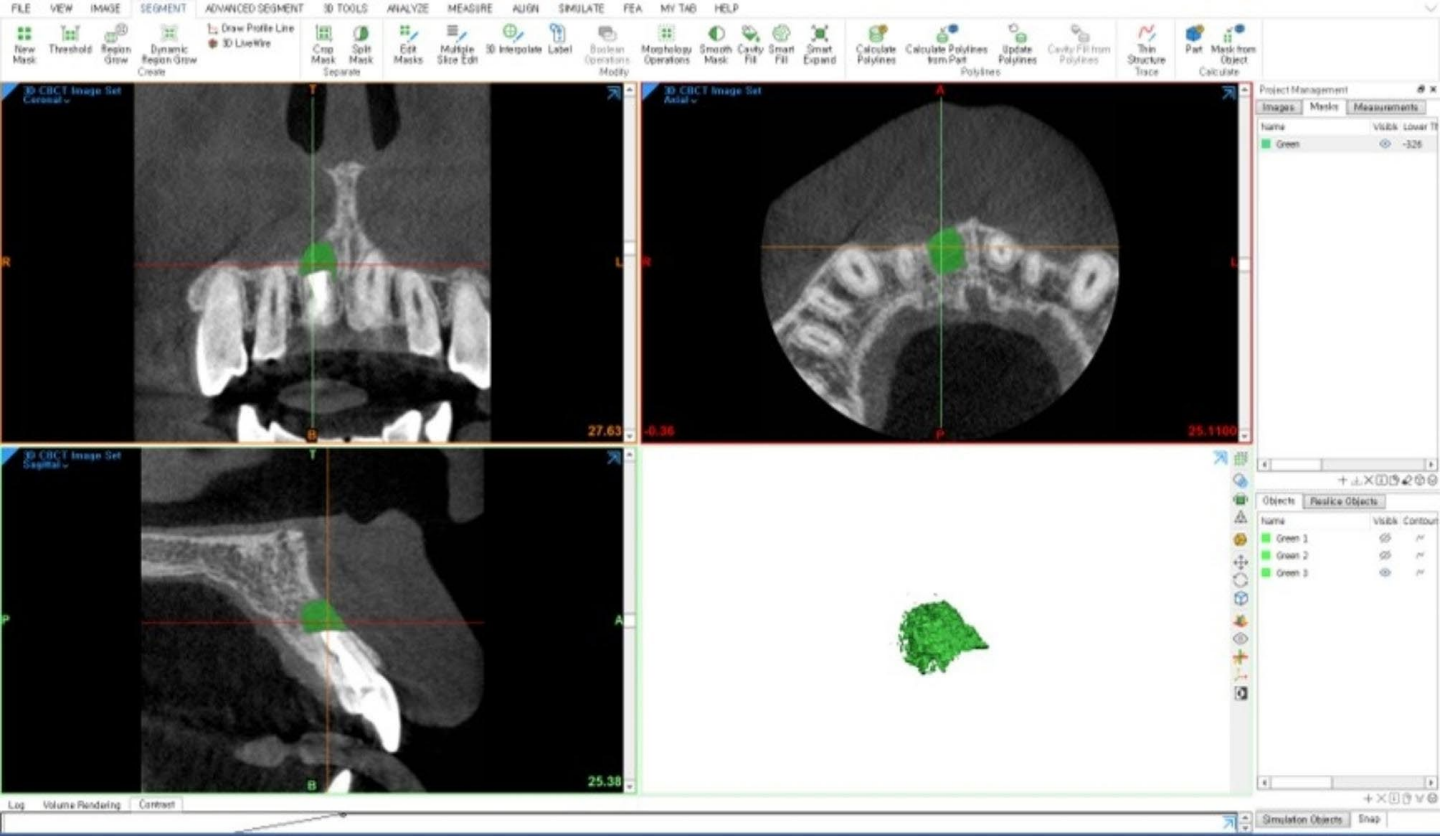
Measurement of the bone-defect volume using Mimics medical software

**Fig. 5:**
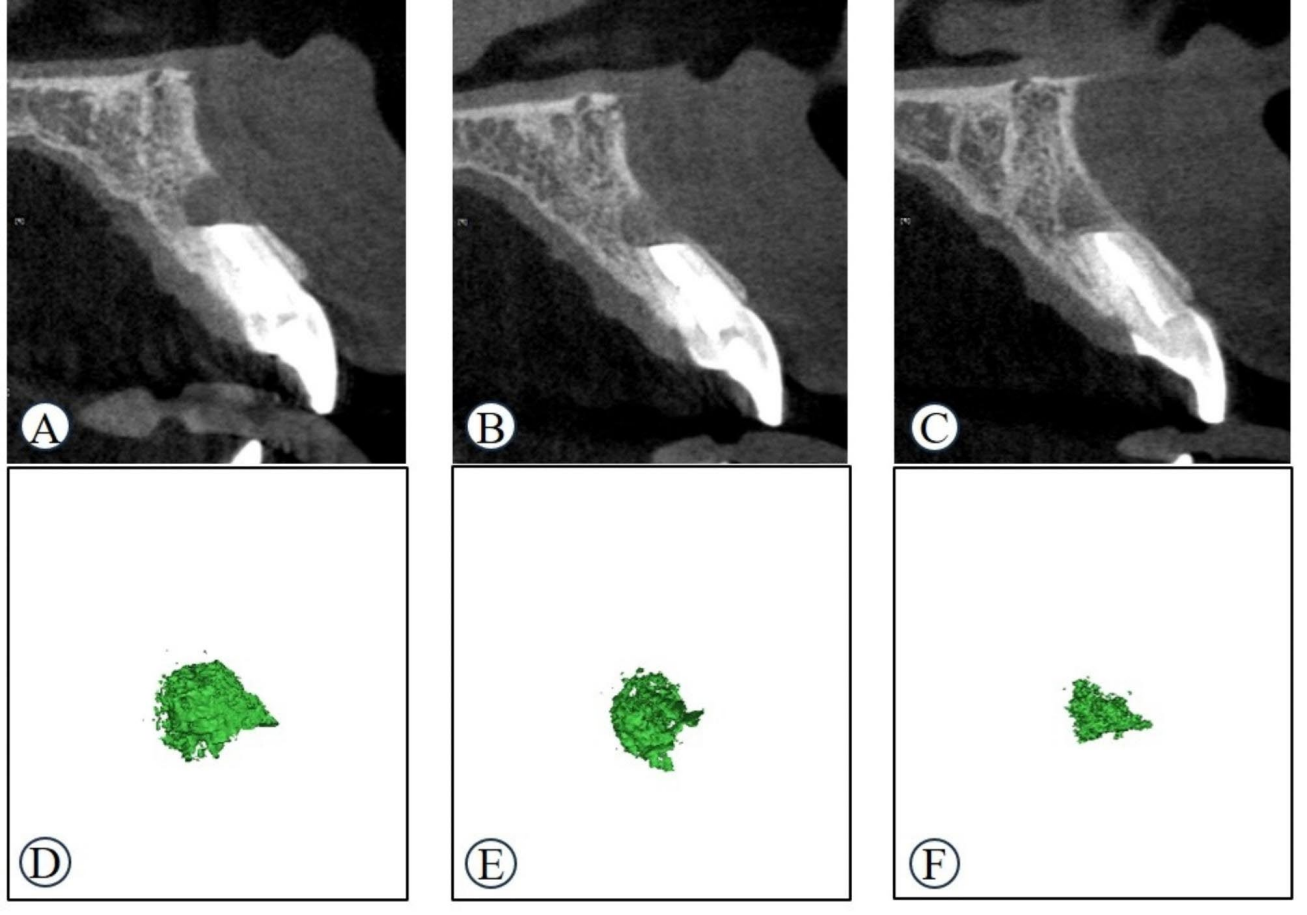
Representative sagittal cone-beam computed tomography and 3D images of the bone defect. **A, D** one week after surgery **B, E** Three months after surgery **C, F** Six months after surgery

To measure the volume of bone defects, CBCT image files in digital imaging and communications in medicine (DICOM) format were transferred to Mimics Medical software version 21.0 (Materialize, Leuven, Belgium). The threshold was appropriately adjusted to mark bone defects. In this study, the standard threshold range was set from − 326 to 670, and a fixed threshold was applied equally to all patients. After image segmentation in the axial, coronal, and sagittal planes, the bone-defect volume was calculated by converting the image into a 3D image (Fig. 4). The bone-defect volume was measured at postoperative one week (V_0_), three months (V_3m_), and six months (V_6m_) (Fig. 5). All volumetric measurements were performed by a single observer (G. J.). The bone-defect volume was measured three times at fortnightly intervals, and the mean bone-defect volumes for all groups at each interval were calculated.

Bone regeneration was compared between the groups based on the rate of volume reduction of the bone defects. As the initial lesion size varied in each patient, the proportion of unfilled lesions was calculated by dividing the mean V_3m_ and V_6m_ by the mean V_0_. The volume reduction rate (%) of the bone defects was calculated by subtracting the proportion of unfilled lesions from 100.

### Statistical analysis

The reduction rate of bone defects was statistically compared between the groups using the Kruskal–Wallis test and post-hoc Mann–Whitney U test with Bonferroni correction. Intraclass correlation coefficients were used to estimate the reliability of volumetric measurements. IBM SPSS software (SPSS 25.0, IBM Corp., NY, USA) was used for statistical analysis. Statistical significance was set at p < 0.05.

## Results

A total of 25 patients (age range; 17 to 68 years, 11 males and 14 females) participated in this clinical trial, and they were randomly assigned to a control group (n = 9) and two experimental groups (PRF group, n = 8 and CGF group, n = 8). Three patients in the control group and two patients in each experimental group failed to recall at follow-up, and eventually, six patients in each group were analyzed (Fig. 1). Table 1 presents the demographic characteristics for each group.

**Table 1:** Demographic data of patients

	Male		Female		
Groups (n = 6)	n	%	n	%	Age (y) (mean ± SD)
control	2	33.33	4	66.67	31.67 ± 17.31
PRF	3	50	3	50	41.83 ± 17.80
CGF	4	66.67	2	33.33	42.67 ± 14.17

SD: standard deviation; PRF: platelet-rich fibrin; CGF: concentrated growth factor

No signs of infection or wound dehiscence were observed, and soft-tissue healing was achieved without complications in all patients at recall follow-ups. The mean V_0_, V_3m_, and V_6m_ are presented in Table 2. The bone-defect reduction rates after three and six months are presented in Table 3. The intraclass correlation coefficient was 0.997, which indicated a high intra-observer consistency and confirmed the reliability of the measured volumes. The bone-defect reduction rate was the highest in the CGF group, followed by that in the PRF and control groups. Compared to one week after surgery, the average bone-defect reduction rate at postoperative three and six months was 44.31 ± 10.5%, 58.85 ± 9.66%, and 60.21 ± 7.40%, and 73.16 ± 17.41%, 78.23 ± 7.52%, and 83.48 ± 6.50% in the control, PRF, and CGF groups, respectively. A comparison of the three groups using the Kruskal–Wallis test revealed a statistically significant difference only at three months after endodontic microsurgery (p < 0.05). After six months, no statistically significant differences were observed between the three groups (p > 0.05). The post-hoc Mann–Whitney U test with Bonferroni correction was performed for multiple comparisons of the bone-defect reduction rate at three months after endodontic microsurgery. Bone regeneration was significantly higher in the PRF and CGF groups than in the control group (p < 0.05). However, no significant difference was observed between the PRF and CGF groups (p > 0.05).

**Table 2:** Bone-defect volumes in each group (Mean ± SD, mm^3^)

Groups (n = 6)	Mean bone-defect volume		
	V_0_	V_3__m_	V_6__m_
control	110.29 ± 70.09	70.78 ± 46.04	39.47 ± 36.40
PRF	246.68 ± 212.81	95.65 ± 83.87	48.72 ± 42.99
CGF	245.27 ± 165.17	95.42 ± 60.32	37.71 ± 27.26

V_0_: volume at postoperative one week; V_3m_: volume at postoperative three month; V_6m_: volume at postoperative six months

**Table 3:** Comparison of the bone-defect reduction rate (%) between groups

	Mean ± SD			*p-value	†Post-hoc		
	control	PRF	CGF		control-PRF	control-CGF	PRF-CGF
3 months	44.31 ± 10.5	58.85 ± 9.66	60.21 ± 7.40	0.000^**^	0.000^**^	0.000^**^	0.548
6 months	73.16 ± 17.41	78.23 ± 7.52	83.48 ± 6.50	0.173			

SD: standard deviation; PRF: platelet-rich fibrin; CGF: concentrated growth factor

*p-value was calculated using the Kruskal–Wallis test

†Post-hoc p-values were calculated using the Mann–Whitney U test with Bonferroni correction

**Statistical significance, p < 0.05

## Discussion

Blood consists of liquid, plasma, and cellular components, including white blood cells, red blood cells, and platelets. Platelets play an important role in hemostasis and contain growth factors involved in angiogenesis and tissue healing. Growth factors are associated with cell migration, proliferation, differentiation, and angiogenesis, and promote the tissue regeneration process [17]. Growth factors include IGF, PDGF, VEGF, and TGF-β, which are abundant in PRF and CGF. IGF is known to form new bones and tissues and regenerate damaged cells by acting on osteoblasts in the endosteum [18]. PDGF exists in platelet α granules or giant cells. This growth factor promotes angiogenesis and osteoblast proliferation. VEGF plays an important role in increasing plasma protein penetration in capillaries, maintaining the survival of new blood vessels, and inducing cell proliferation and differentiation [19]. TGF-β affects osteoblasts at an early stage of development and fibroblast to stimulate collagen synthesis promoting bone and cartilage regeneration [20]. In addition, PRF and CGF contain pro-inflammatory cytokines such as IL-6, IL-1β, and TNF-α, as well as the anti-inflammatory cytokine IL-4 [21]. As previously mentioned, PRF and CGF with three-dimensional fibrous networks allow for the release of these growth factors slowly over 7–14 days [22]. This suggests that PRF and CGF may inhibit postoperative inflammation and contribute to the early stages of bone formation. Therefore, blood components have been used to promote healing in dentistry, and studies have been conducted.

Several studies have reported the effects of PRF and CGF on bone regeneration. Kim et al. [23] showed that bone mineral density and volume were high when PRP, PRF, or CGF were applied to rabbits with cranial bone defects. However, in a study by Knapen et al. [24], PRF did not appear to have any additional effect on the quality, quantity, or kinetics of guided bone regeneration. Several in vivo and in vitro studies on PRF and CGF have been conducted, but the effectiveness of PRF and CGF remains controversial.

Various clinical studies have been conducted on bone regeneration using PRF and CGF. In implant surgery, PRF and CGF provide stabilization of the bone graft in the defect area and minimize bone loss during healing [25]. PRF and CGF have been shown to reduce the soft-tissue healing time, postoperative pain, edema, and trismus after third molar extraction [26, 27]. Another study reported that PRF significantly reduced pocket depth and improved the clinical attachment level in intrabony periodontal defects [28]. Lei et al. [29] showed that advanced PRF and CGF improve the outcome of guided tissue regeneration by stimulating the steady release of growth factors.

Unlike in other fields, reports on the effects of PRF and CGF in endodontic microsurgery are scarce. Studies evaluating the clinical effect of PRF alone on bone regeneration after periapical cyst enucleation reported that PRF promotes faster bone regeneration within three months after surgery, and complete bone regeneration and bone density are observed six months after surgery [13, 30]. This was similar to the result that bone regeneration was promoted at postoperative three months in the PRF group in this study. Dhamija et al. [31] reported better healing when PRP, but not PRF, was applied to through and through lesions. However, Dhiman et al. [32] reported no significant effect when PRF was applied to apicomarginal defects. Unlike the previous two studies, this study used PRF and CGF in small defects (≤ 10 mm) and showed good results with regard to early bone healing. Another difference was the use of platelet-derived materials. This study used PRF and CGF in the form of a membrane, which is the same method as in Dhiman et al.’s study [32]; however, in other studies, PRP was packed into the defects [13, 30]. No study has evaluated the differences according to the application method after endodontic surgery, and further studies are needed to determine whether there is a difference in the effects depending on the application method.

Several studies on healing after endodontic microsurgery has used traditional two-dimensional periapical radiographs [33]. However, two-dimensional analysis has some limitations such as superimposition and distortion. And it is difficult to take radiographs at the same position and condition at follow-up visits for comparing bone healing. CBCT analysis allows for a more precise evaluation of periapical lesions than periapical radiographs [34]. In this study, the bone-defect volume was analyzed in three dimensions using CBCT for accurate evaluation, and through quantitative analysis, it was possible to evaluate early bone healing that was difficult to distinguish in two-dimensional radiographs. Since bony structures, including the labial cortical bone, were removed during surgery, the measurement at postoperative one week, not the preoperative measurement, was used as the baseline value for accurate comparison of bone healing in this study.

In this study, new bone formation after three months was more in the experimental groups than that in the control group, demonstrating that PRF and CGF promote bone regeneration. However, no statistically significant difference was observed between the PRF and CGF groups. These results demonstrate that platelet-derived materials are effective for stimulating new bone formation. In the evaluation of bone defects after six months, no significant differences were observed between the three groups. Kim et al. [35] reported that the volume of the preoperative periapical lesion significantly affected the outcomes of endodontic microsurgery, and it is thought that there was no significant difference in bone regeneration after six months because the size of the lesions was small.

Attempts to use PRF with bone grafts (sticky bones) have also been reported [36]. In this study, no graft material was used for bone regeneration because the use of bone-graft material hinders the measurement of the volume of new bone [37]. Another method of use has been proposed to use PRF as a natural carrier for antibiotic application [38]. This is known to have the effect of reducing post-surgical infection through effective antibiotic application, and such attempts need to be studied further in surgical endodontics.

This study had some limitations. First, the number of participants was relatively small. Second, the age or sex of the patients was not taken into consideration. These factors may affect the action of growth factors or healing status. Third, the sizes of the bone defects were limited. Fourth, only the effects over a relatively short period of six months were evaluated. Thus, future studies are required in patients with larger and similar-sized lesions with long periods of follow-up.

## Conclusions

According to the results of this study, the first hypothesis that PRF and CGF promote early healing of bone defects after endodontic microsurgery was accepted. And the second hypothesis that there is no difference between PRF and CGF in promoting early healing of bone defects after endodontic microsurgery was also accepted. Within the study limitations, this study clinically demonstrated that PRF and CGF have a positive effect on early bone formation and may enhance the therapeutic effectiveness of endodontic microsurgery. Large-scale prospective clinical studies are required to further evaluate the possible benefits of PRF and CGF in endodontic microsurgery.

## Data Availability

The data presented in this study are available on request from the corresponding author.

## Abbreviations

PRF: platelet rich fibrin
CGF: concentrated growth factor
CBCT: cone-beam computed tomography
IGF: insulin-like growth factor
PDGF: platelet-derived growth factor
VEGF: vascular endothelial growth factor
TGF: transforming growth factor
